# Modified silicas with different structure of grafted methylphenylsiloxane layer

**DOI:** 10.1186/s11671-016-1513-7

**Published:** 2016-06-13

**Authors:** Yuliia Bolbukh, Konrad Terpiłowski, Roman Kozakevych, Dariusz Sternik, Anna Deryło-Marczewska, Valentin Tertykh

**Affiliations:** Chuiko Institute of Surface Chemistry of National Academy of Sciences of Ukraine, 17 General Naumov Str., Kyiv, 03164 Ukraine; Maria Curie-Sklodowska University, Sq. Maria Curie-Sklodowska 2, Lublin, 20-031 Poland

**Keywords:** Nanodispersed silica, Surface modification, Polymethylphenylsiloxane (PMPS), Dimethyl carbonate (DMC), Chemical assembly approach, Surface layer structure, Hydrophobicity, 68.43.Hn, 81.07.Wx, 81.16.Be

## Abstract

The method of a chemical assembly of the surface polymeric layer with high contents of the modifying agent was developed. Powders of nanodispersed silica with chemisorbed polymethylphenylsiloxane (PMPS) were synthesized by solvent-free chemical assembly technique with a dimethyl carbonate (DMC) as scission agent. Samples were characterized using FTIR spectroscopy, transmission electron microscopy (TEM), atomic force microscopy (AFM), and elemental analysis (CHN analysis). Coating microstructure, morphology, and hydrophilic-hydrophobic properties of nanoparticles were estimated. The results indicate a significant effect of the PMPS/DMC ratio at each modification stage on hydrophobic properties of modified silicas. Modification with a similar composition of the PMPS/DMC mixture, even with different polymer amount at each stage, provides the worst hydrophobicity. Results suggest that the highest hydrophobicity (contact angle *θ* = 135°–140°) is achieved in the case when silica modified with the PMPS/DMC mixture using multistage approach that providing a formation of the monomolecular layer of polysiloxane at the first modification step. The characteristics of surface structure were interpreted in terms of density of polymer-silica bonds at the interfaces that, usually, are reduced for modified surfaces, in a coupling with conformation model that accented the shape of chains (arch- and console-like) adsorbed on solid surfaces.

## Background

The hydrophobic nanodispersed silicas are widely used as fillers for adhesives and sealants, epoxy resins, vinylester resins, gelcoats, cable gels, and natural and artificial oils and grease, as well as component of biological dispersions and sorbents. The most promising among the above materials are polyorganosiloxane-modified pyrogenic silicas which are characterized by thermal stability, chemical inertness, and hydrophobic properties [1–3]. These properties are determined by a type of organosilicon polymer/silica interaction, a thickness and architecture of the grafted surface layer.

The functionalization of silica nanoparticles with polymers requires providing a sufficient amount of surface contacts with agglomeration being prevented and high dispersity being maintained in order to serve the advantages of a nanosized product. Methods of nanoparticles modification with polyorganosiloxanes include several common approaches, namely, an adsorption modification from solvents with follow stitching of immobilized layer by radiation [4], or using a crosslinking agent [2], as well as thermal treatment of the adsorbed polysiloxane layer at 200–300 °C [5, 6]; the modification with the monomer while its polymerization is carried out, and gas-phase polymer immobilization under inert atmosphere and the modifying agent vapor pressure (300–400 °C) [7]. The polymeric chain attaching is realized via interaction of polymer functional groups with native surface groups [8] or chemically active groups grafted onto the silica surface [9, 10]. It should be noted that the modification in a gas phase allows one to maintain silica particles dispersity; however, the obtained composites are characterized by a low content of the modifying agent attached.

One of the efficient methods to obtain the modifying layer with high content of polymer on the silica surface is a chemical assembly of the grafted layer that is realized by growing the polymer with the specified structure from the monomers and oligomers [11, 12]. On the other hand, grafted polymer layer of the desired structure can be formed via an activation of the polysiloxane by a depolymerization of macromolecules with further grafting of obtained oligomers on the silica surface. Depolymerization can be carried out thermally (300–400 °C) [5] or by treatment with active agents such as alkalis, hydrochloric or sulfuric acids [13], thionyl chloride [14], amines [15], or a mixture of alkali (NaOH, KOH) with alcohols (methanol, ethanol) [16], as well as dimethyl or diethylcarbonate [17], which does not introduce contaminants into the system.

Aside from chemical or thermal activation of polymeric modifying agent, a procedure of modification is important for producing the attached surface layer with desired structure. Namely, different amount of polysiloxane in hexane solution at fumed silica impregnation caused a different structure both the attached polymeric layer and the nanoparticles aggregates [18]. Other method of chemical assembly of the surface polymeric layer with high contents of the modifier was proposed in [19]. The main approach was regarded to adsorption of polydimethylsiloxane from dilute solutions in dichloromethane that was carried out stepwise from solution with different concentration. It was found that the less amount of the modifying agent is absorbed if the particles surface was pretreated with the solution of the polymer at the lower concentrations. Higher concentration of grafted macromolecules was achieved within a fixed time (week) with increasing concentration of the polydimethylsiloxane in the modifying solution.

The structure of the attached polymeric layer can be controlled by silica modification [20]. More promising is a combination of the silica or modificator activation with the multistep process. As was shown in [21] the “stepwise growth” method of the silica modification by oligomers with different amount of chemically active groups allows one to obtain comb-branched and dendritic-branched polymers.

Scale-up synthesis of polymer-grafted inorganic nanoparticles in a solvent-free dry system attracts great attention for prevention of environmental pollution and simplification of reactions. Grafting of polymers in a solvent-free dry system was achieved by monomer spraying onto nanoparticles having initiating groups [22]. Along with the solvent-free dry systems, “green” reagents, particularly dimethyl carbonate (DMC) [17], gained wide propagation.

The present paper concerns to synthesis and investigation of the polysiloxane-modified silica with a fine dispersity and high modification degree. The investigation is aimed to elaborate a new method to control a structure of a polysiloxane layer chemisorbed on silica surface and to investigate an influence of applied methodology on hydrophobic properties of modified silicas. For realizing the assigned task, a solvent-free chemical assembly technique that combines a depolymerization reaction at dimethyl carbonate action and the chemisorption of oligomer formed was proposed. The advantages of the proposed method are the solvent-free process and using “green” chemistry reagents in comparison with state-of-art in area. The proposed approach not only can ensure the cleanliness of the graft layer and the control of its structure but also may facilitate in reducing the temperature of the modification process.

### Background of the Study

Thermal depolymerization of polydimethylsiloxanes has been well studied. It is known that at temperatures above 300 °C, the formation of cyclic oligomers is observed. A major fraction is represented by cyclic trimer (–SiR_2_–O)_3_, although there are tetramers, pentamers and other oligomers with a larger number of siloxane units. The strict scission of the macromolecules due to the helical structure of polymeric chains and the bending degree of the main helix defines the number of the siloxane bridges in species eliminated during thermal decomposition. Most probable mechanisms of oligomers elimination from polysiloxane molecules are cyclization and scission with the involvement of the terminal hydroxyl groups and intramolecular splitting of the polymeric chain (elimination of loops) [23].

**Scheme 1.**
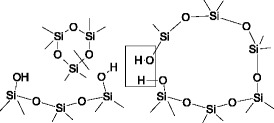
Thermal depolymerization of polyorganosiloxane

The interaction of polyorganosiloxane with functional groups of the silica surface during thermal depolymerization, in particular in fluidized bed, can be represented by Scheme 1.

Depending on the thermal depolymerization conditions, the modifying layer may be comprised of the loops and free oligomeric chains with the terminated (secondary) silanols at the ends.

The use of dimethyl carbonate as a scission or depolymerization agent for polysiloxane was first proposed by the authors of [24]. They have studied depolymerization of polydimethylsiloxane (PDMS) and polymethylphenylsiloxane in the presence of DMC in the methanol solution with the addition of alkali metal halides as catalysts.

**Scheme 2.**
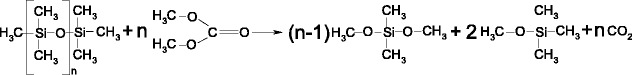
Interaction of dimethyl carbonate with polydimethylsiloxaneᅟ

The authors proposed the following reaction scheme (Scheme 2).

The product of reaction which was carried out at 180 °C with a ratio of PDMS/DMC/methanol as 1.5/1.8/7 g is mainly dimethyldimethoxysilane (70 %). Authors have pointed out that methanol as well as potassium fluoride had the most important action on depolymerisation reaction [25].

According to [26], the depolymerisation of polymethylphenylsiloxane by action of dimethyl carbonate occurs at the ends of polymeric macromolecules, the main process is completed in 30 min, and the depolymerization products were dimethyldimethoxysilane (about 70 %), trimethylmethoxysilane (10 %), and methylphenyldimethoxysilane as well as disiloxanes with methyl and phenyl groups. It was shown that the increase in the yield of low molecular weight products is ensured by the presence of alcohol.

An important role of alcohol in the depolymerisation reaction was marked also by authors of [16]. It was found that at polysiloxane depolymerisation using a mixture of potassium hydroxide in ethanol and diethylamine the oligomers with higher molecular weight are formed in the absence of alcohol. It is shown that alcohol molecules play a role as a solvent and nucleophile. The active site in the reaction of siloxane bridges scission by alcohols is alkoxide ion. Typically, alcoholysis is base-catalyzed reaction, but in case of orienting alcohol molecules near the siloxane chains, it can be initiated thermally. The reaction of the siloxane bond splitting by alcohols has first order and in the general form it can be written as follows [26, 27]:

$$ {\left(\mathrm{C}{\mathrm{H}}_3\right)}_3\mathrm{S}\mathrm{i}-{\left[\mathrm{O}-\mathrm{S}\mathrm{i}{\left(\mathrm{C}{\mathrm{H}}_3\right)}_2\right]}_{\mathrm{n}}\hbox{--} \mathrm{O}\mathrm{S}\mathrm{i}{\left(\mathrm{C}{\mathrm{H}}_3\right)}_3 + 2n\mathrm{C}{\mathrm{H}}_3\mathrm{O}\mathrm{H} = \left(n- 1\right){\left(\mathrm{C}{\mathrm{H}}_3\right)}_2\mathrm{S}\mathrm{i}{\left(\mathrm{O}\mathrm{C}{\mathrm{H}}_3\right)}_2 + 2{\left(\mathrm{C}{\mathrm{H}}_3\right)}_3\mathrm{Si}\mathrm{OC}{\mathrm{H}}_3 + n{\mathrm{H}}_2\mathrm{O} $$

**Scheme 3.**
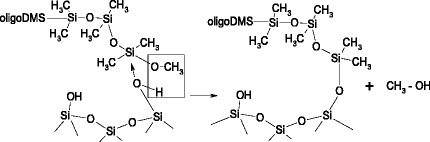
Esterification of silanol groups on the silica surfaceᅟ

**Scheme 4.**
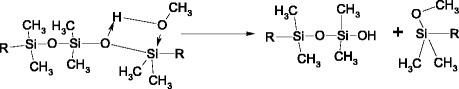
Esterification of siloxane bonds of the organosiloxaneᅟ

The alcohol provides formation of alkoxysilyl groups at the ends of macromolecules, which are less stable due to weak hydrolytic resistance of Si-O bonds that leads to cyclization (or crosslinking) of oligomers. Such groups are chemically active with respect to the silica surface hydroxyl groups and provide chemisorption of polysiloxanes [26, 27]. During interaction of methoxy terminated oligomers with the silica surface hydroxyls produced methanol (Scheme 3) that can react with siloxanes not only with the formation of methoxyl groups and water but also according to the Scheme 4.

**Scheme 5.**
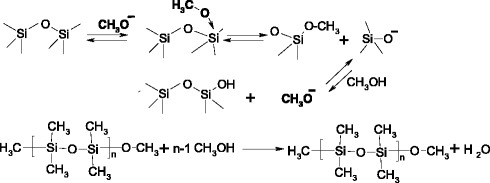
Branching of the polysiloxane backbone

Thermal decomposition of methyl groups at silicon atom starts at 220–300 °C, but in the presence of alcohol (namely, methoxy groups), they decompose even at 180 °C and the resulting alkoxy-radical can again react with the siloxane bond (Scheme 5) [27].

**Scheme 6.**
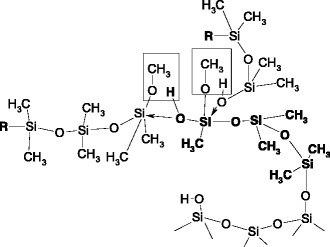
Creation of a polysiloxane layer on the silica surfaceᅟ

The condensation of hydroxyl and methoxy groups at the ends of the oligomers can provide branched macromolecules backbone that can be used for stepwise modification of the silica surface by the chemical assembly method (Scheme 6).

Thus, the following assumptions are the basis of this study. Upon the silica surface modification with polymethylphenylsiloxane (PMPS) using depolymerisation reaction in the presence of the dimethyl carbonate for polymer activation:

1. The products of depolymerization are linear oligomers and the depolymerization degree is determined by the concentration of dimethyl carbonate [28, 29];
2. The presence of methoxy groups promotes grafting of polymers on the silica surface. Formation of several methoxy groups at the oligomer ends allows one to carry out the chemical assembly reaction;
3. Methanol eliminated during depolymerization allows one to conduct the modification reaction without solvent, besides, the methanol interaction with the polymeric chains promotes the formation of hydroxyl group which exhibit a high reaction activity with respect to both the silica surface and the methyl groups at the ends of the oligomers. In addition, the presence of alcohol can reduce the temperature of thermal depolymerization, and hence can cause a crosslinking of the grafted layer.

It was assumed that the change in the polymer/dimethyl carbonate ratio at different stages of modification process allows one to assemble surface layer with different structure. The grafting of the polymer was conducted by multistage chemical assembly technique using solvent-free modification in fluidized bed. The objectives were to investigate the effect of the oligomers size and amount of modifying agent on the modification efficiency at each stage, as well as the influence of these parameters on the structure of the layer attached.

Modification of silica surface was performed with mixtures with different ratios of the organosilicon polymer and initiator at each stage. Different compositions of modifying solutions and an order of the component introducing into reactor should result in the formation of surface layers with different chain length of the oligomers attached. Chain length is determined by the DMC content in the modifying mixture. Two approaches were applied: modification “from long to short”—when the DMC content in modifying mixtures is increased from stage to stage, or “from short to long”—DMC content is decreased for the each following stage. In the latter case after the first modifying step, there is a possibility to form dendrimer-like surface layer.
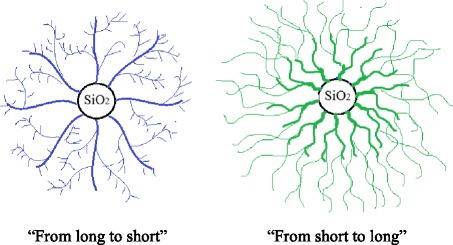


In the case of the “long-chain” oligomers attaching at the first stage, the next stage is realized via the interaction of the modifying mixture with immobilized polymer hanging loops, which causes their scission. The opposite approach mainly promotes the formation of linear grafted oligomeric structures. It is expected that a hierarchical structure of the grafted surface layer will exhibit high water-repellent properties. So, this investigation is devoted to the study of nanodispersed powders obtained by chemisorption of polysiloxane on silica surface using a chemical assembly technique.

## Methods

The study was performed using fumed silica with specific surface area of 260 m^2^/g (A-300, Kalush, Ukraine). PMPS fluid (Zaporizhzhya, Ukraine, PMPS–4, GOST 15866-70) was used as a modifying agent. DMC from Sigma-Aldrich was used as a depolymerisation agent.

### Analytical Technique

In order to control the passage of surface reactions, infrared spectra were recorded using a ThermoNicolet FTIR spectrometer with a diffuse reflectance mode or a Specord M-80 (Carl Zeiss) spectrometer with a transmittance mode in a range of wavenumbers 4000–200 cm^−1^. The silica samples were pressed into rectangular 28 × 8 mm plates of 25 mg weight.

To measure the content of grafted organic groups in the synthesized samples the Perkin-Elmer 2400 CHN-analyzer (USA) was used. The modifying layer was oxidized to produce H_2_O and CO_2_ during the samples heating in the oxygen flow at 750 °C. The structure of grafted layer was analyzed by X-ray photoelectron spectroscopy using a spectrometer XPS/UPS: system analytic UHV, Prevac. Coating microstructure and morphology of the modified samples were analyzed using transmission electron microscopy (TEM) (Tecnai G2 T20 X-TWIN, USA). Specific surface area was measured using nitrogen adsorption-desorption data recorded with the help of Accelerated Surface Area and Porosimetry analyzers ASAP 2020 and 2420 (Micromeritics, USA). Before measurement the degassing of samples was performed followed analysis was carried out according with ASAP 2020 Physisorption Laboratory Practical Exercise Guide [28]. The particle morphology was studied using atomic force microscopy (AFM) (NanoScope III, Digital Instruments, USA, with a tapping mode AFM measurement technique).

Hydrophilic-hydrophobic properties of the surface of the obtained modified silicas were estimated by measurements of contact angles of wetting. Advancing (ACA) and receding (RCA) contact angles of water drops supported on pressed (180 bars for 15 min) plates of samples were measured using a GBX contact angle meter (France) equipped with a closed and humidity-controlled measuring chamber and a digital camera. The measurements were carried out at 20 °C and 50 % relative humidity. To measure ACA, 6 μl water droplet was gently settled on the sample surface using an automatic deposition system and contact angles were evaluated for both sides of the sample plate using the Win Drop++ program. Then, 2 μl of the droplet volume was sucked into a syringe and RCA was estimated. To obtain the averaged ACA and RCA values, the measurements were performed for ten water droplets supported on the each sample. The apparent surface free energy (*S*) was calculated from the ACA and RCA values using a hysteresis approach [30]. The roughness and topography was investigated by light scattering spectroscopy using an optical profilometer (Contour GT-K1, Veeco). The apparatus is equipped with an optical surface-profiling system (3D) measuring surface morphology with high accuracy from sub-nanometer up to 10 mm size. Measurement was carried out after powders compressing into plates.

### Modification of Silica Surface

Nanodispersed fumed silica was modified with PMPS in a three-stage process using DMC as an initiator of the polymer chain scission. The process was carried out in a laboratory batch reactor (2 l) after air and moisture removing by blowing nitrogen gas at stirring and heating at 200 °C for 1 h. Before introducing reagents, the reactor with pretreated silica (10 g) was closed and the silica surface modification was performed in a gas nitrogen atmosphere. The fluidized bed was formed by overhead stirrer. Modification was performed with the PMPS/DMC mixture with the different ratios of polymer and initiator (3.4, 5.8, 20.0) (Table 1) and amount of the modified mixture at each stage (Table 1). The optimal reaction temperature was 250 °C, the polymer content for all composites was 20 wt% of silica mass, and the total amount of dimethyl carbonate was 18 % of PMPS volume.

**Table 1:** The modified mixture portions (vol%) and PMPS/DMC ratio at each stage of the preparation of silica/PMPS composites applying chemical assembly approach

Sample no.	Total PMPS/DMC ratio	PMPS cont., wt %	Modification scheme	PMPS/DMC ratio in the modified mixture			Portion of modified mixture, vol%		
				Modification stages			Modification stages		
				I	II	III	I	II	III
Protocol 1									
1	5.5	20	L-S	20.0	5.8	3.4	25	35	40
2	5.5	20	M	5.5	5.5	5.5			
3	5.5	20	S-L	3.4	5.8	20.0			
Protocol 2									
4	5.5	20	S-L	3.4	5.8	20.0	40	35	25
5	5.5	20	M	5.5	5.5	5.5			
6	5.5	20	L-S	20.0	5.8	3.4			

As represented in Table 1, the modification was carried out using two protocols. At *protocol 1*, the modifying mixture PMPS/DMC was sprayed into the reactor by portions of 25, 35, 40 vol% of total amount on each stage. According to *protocol 2*, the modifying mixture was introduced into the reactor by the portions of 40, 35, 25 vol%. The chemical assemble of polymer was carried out using the oligomers with different chain length controlled by the ratio of polymer (PMPS) and depolymerisation agent (DMC) at each stage of modification (Table 1). According to one scheme (“from short to long” or “S-L”) at the first stage, the mixture with high DMC content (as compared to other stage) was introduced into the reactor. As expected, high content of depolymerisation agent promotes a higher degree of polymeric chain scission. Under these conditions, “short-length” oligomers would immobilize first. At the next stage, the DMC content in the mixture was reduced in order to obtain “middle-length” oligomers. At the last modification stage, the PMPS/DMC ratio was lowest in order to obtain “long-chain” oligomers. According to the scheme “from long to short” (L-S) at the first stage, the mixture with the lowest DMC content was introduced into the reactor. At next stages, the amount of DMC was increased. This approach would promote immobilization of “long-chain” oligomers on the silica surface during the first modification stage. The scheme with the same polymer/DMC ratio at each stage was defined as “middle” (M). The modifying mixture was prepared before the each stage. The modification process was carried out under inert atmosphere (gas N_2_) for 2 h. Before modification, the silica sample was heated at 150 °C for 1 h in N_2_-gas flow.

## Results and Discussion

Table 1 represents compositions and amount of the modified mixture, which was applied for powder synthesis at each stage of chemical modification.

**Fig. 1:**
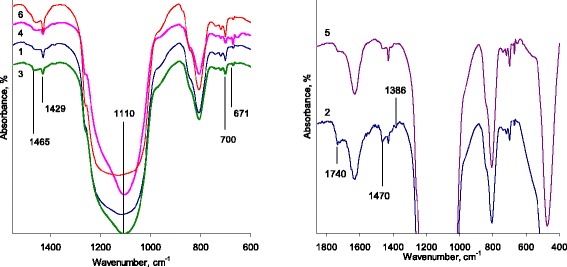
FTIR spectra of silicas with attached PMPS (samples 1–6) using different modification protocols and scheme (Table 1). The illustrations show the IR spectra of composites obtained by protocol 1 (samples 1–3, vol% of the modifying mixture at each stage 25/35/40) and protocol 2 (samples 4–6, vol% of the modifying mixture at each stage 40/35/25). The spectra for samples 2 and 5 are located according to the quantity of the modifying mixture at the first stage when the ratio of PMPS/DMC in the modifying mixture at different stages of modification was equal to 5.5

FTIR-spectra of samples (Fig. 1) pointed on less difference in attached layer except spectra region of structure-sensitive bands 760–550 cm^−1^*.* For composite *1*, the band at 671 cm^−1^, attributed to –CH= bond in benzene ring, was less defined. Also, for this composite, the change in the shape of the band assigned to Si–O–Si bonds is caused by changes in the polymer/surface interaction. For composite *2*, at less amount of modifying mixture comparing to composite *5*, the presence of carbonyl or ethoxyl group (1740 cm^−1^) at surface layer is detected. The new band at 1386 cm^−1^, corresponding to methyl group deformation vibration, may be due to the presence of Si–O–CH_3_ bonds.

**Fig. 2:**
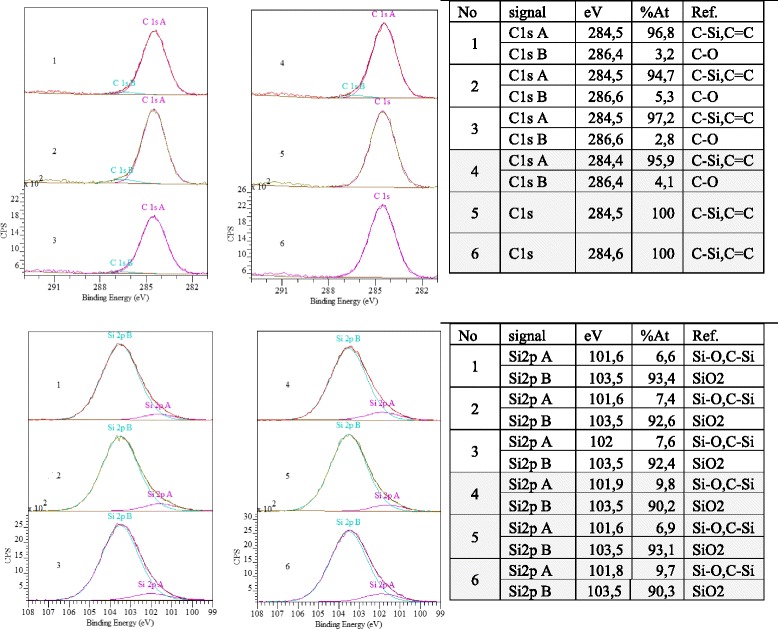
XPS spectra of silica modified with PMPS using different synthetic protocols. Spectra present the changes in binding energy C1s and Si2p for composites of silica modified with PMPS using protocol 1 (samples 1–3, vol% of the modifying mixture at each stage 25/35/40) and protocol 2 (samples 4–6, vol% of the modifying mixture at each stage 40/35/25) by chemical assembly process in the presence of dimethyl carbonate at PMPS/DMC ratio according to the schemes “L-S” (samples 1 and 6), “M” (samples 2 and 5) and “S-L” (samples 3 and 4) as represented in Table 1

The XPS patterns (Fig. 2) show that the grafted layer is different in general. The carbon content in the samples under study was approximately 11 wt%, except sample 3, in which the carbon content was 8.9 wt% (Table 2).

**Table 2:** XPS results for silicas modified with polymethylphenylsiloxane

Sample	Peak	Binding energy, eV	Concentration		Sample	Peak	Binding energy, eV	Concentration	
			at %	wt %				at %	wt %
1	C1s	284.0	17.3	10.5	4	C1s	285.0	18.4	11.2
	O1s	532.5	45.0	36.3		O1s	533.0	44.1	35.7
	Si2p	103.5	37.7	53.2		Si2p	103.5	37.5	53.2
2	C1s	284.5	18.4	11.2	5	C1s	284.0	18.1	10.9
	O1s	532.5	44.9	36.4		O1s	532.5	44.5	36.0
	Si2p	103.5	36.7	52.4		Si2p	103.5	37.4	53.1
3	C1s	284.0	14.9	8.9	6	C1s	284.5	18.1	11.0
	O1s	533.0	47.0	37.6		O1s	532.5	44.9	36.3
	Si2p	103.5	38.0	53.4		Si2p	103.5	37.1	52.7

Analysis of the XPS spectra was performed using the Gaussian model. The C1s peak is split into two components with binding energies of 284.4 eV (C=C together with Si–C bonds) and 286.5 eV (C–O bond) (Fig. 2). The peak at 286.4–286.6 eV can indicate the presence of alcohol formed at the polymer immobilization in the presence of dimethyl carbonate.

For samples *5* and *6*, the C1s peak is satisfactorily described by a single band with extremum at binding energy of 284.5 eV. The Si2p peak is described by two components with binding energy of silicon in the silica structure at 103.5 eV and siloxane bridges Si–O (O–Si–C) at 102–101.8 eV. The position of this peak varies depending on the nature of the organic substituent near main chain. The change in an intensity of the peak at 101.6 eV attributed to the binding energy of silicon in the polysiloxane chain (Fig. 2) was marked in peak of the silicon (Si2p) for samples *5* and *6*. This may indicate a different effect of surrounding groups on the O–Si–O bridges and hence a different mutual orientation of the molecules in the grafted layer. At the same time, the carbon contents for samples *5* and *6* are almost equal (Table 2). For sample *3*, an extremum of the peak that corresponded to siloxane bonds is observed at 102 eV, which is slightly higher than binding energy *E*_b_ for other samples. Changing in the binding energy is associated with the amount and nature of substituents at silicon atom (shifts toward large *E*_b_ values indicate a decrease in the amount of Si–C). Changes in the spectrum of the silicon with the intensity variation of the peak at 286.6 eV can indicate differences in the ratio of bonds O–Si–C, C–O–Si and Si–CO, in comparison with other samples.

According to the literature [29], the binding energy of 532.5 eV relates to hydroxide (Si–O–H or C–OH in Si–O–Si or Si–OC links). The shift of this band to higher energies is usually due to the presence of adsorption interaction (namely, water sorption on silica surface), and for siloxanes—increased contribution to the overall range of groups with bonds Si–O–C as a consequence of the formation of new carbon-containing group.

Since the spectrum O1s for samples *3* and *4*, there is no evidence of adsorbed water presence, the contribution of the groups with a binding energy of about 286.5 eV is different (except identical increase in the contribution of the C–O into the total spectrum). The same change in binding energy (with respect to all other samples) is due to an increase in interaction between the chains of the grafted polymer. Although for the sample *3*, increase in oxygen content with reducing of the carbon content may indicate the formation of secondary Si–OH groups instead of Si–C–OH.

**Fig. 3:**
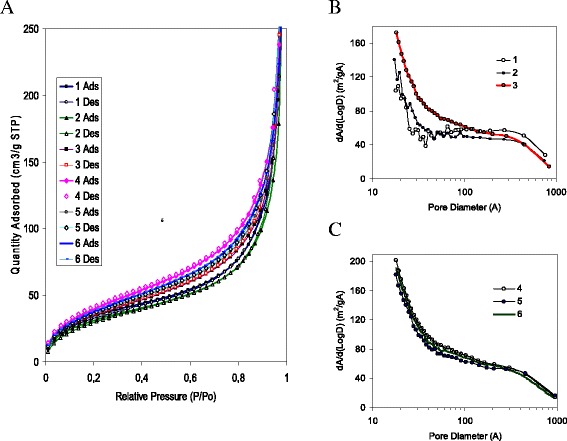
Nitrogen adsorption isotherms (**a**) and pore size distribution for silicas modified with PMPS using protocol 1 (**b**) and protocol 2 (**c**). Figures illustrate nitrogen adsorption on silicas modified with PMPS using multistage process with different ratios of polymer and depolymerization agent (PMPS/DMC) with introducing at the first stage the short- (samples 3 and 4), middle- (samples 2 and 5), and long-length oligomers (samples 1 and 6) and application of two protocols of polymer chemisorption. The pore size distribution was calculated from desorption branch of isotherm for samples synthesized using protocol 1 (samples 1–3) and protocol 2 (samples 4–6) (see Table 1)

To analyze the structural characteristics the low-temperature (77.4 K) nitrogen adsorption–desorption isotherms were recorded (Table 3). The resulting isotherms (Fig. 3a) are typical for such materials [30]. For nonporous materials the presence of hysteresis is caused by textural porosity of the powders (Fig. b and c) responsible for the distance between the particles or aggregates of particles [31]. For this type of powder, specific surface area does not increase due to the rise of material porosity but depends on the degree of availability of a surface and its capacity to the nitrogen adsorption. For instance, the specific surface area (*S*) and the adsorption capacity (*V*_p_) for nitrogen depends on compacting of primary particles in aggregates and agglomerates of aggregates, as well as other parameters characterizing the contributions of nanopores (*D* < 1 nm), mesopores (1 < *D* < 50 nm), and macropores (*D* > 50 nm) (Table 1), being varied at the different modification protocols. For the test samples, the pore size, calculated from desorption branch of the isotherm by BJH, is within the range of 14–20 nm and corresponds to mesopores.

**Table 3:** The specific surface area and textural characteristic (pores volume and pores diameter) estimated by N_2_ adsorption-desorption isotherms

No.	*S*, m^2^/g			Pore size, Å		Pore volume, cm^3^/g
	BET	BJH	*t*-plot	BET	BJH	BJH des.
1	126	105	118	195	223	0.584
2	118	103	122	182	199	0.510
3	133	118	152	146	155	0.459
4	154	139	179	151	159	0.551
5	139	129	163	173	178	0.575
6	146	131	171	139	146	0.479

The specific surface area of silicas after modification is reduced compared to the pristine nanosilica (Table 3). According to [30], a decreasing *S* value could depict diminution of aggregation of primary particles in secondary one, i.e., the size of aggregates becomes smaller, which results in an increase in the bulk density of agglomerates. For instance, individual primary particles are observed in SEM and AFM images of silica with lower *S* in contrast to other nanosilicas with higher *S* values. .

**Fig. 4:**
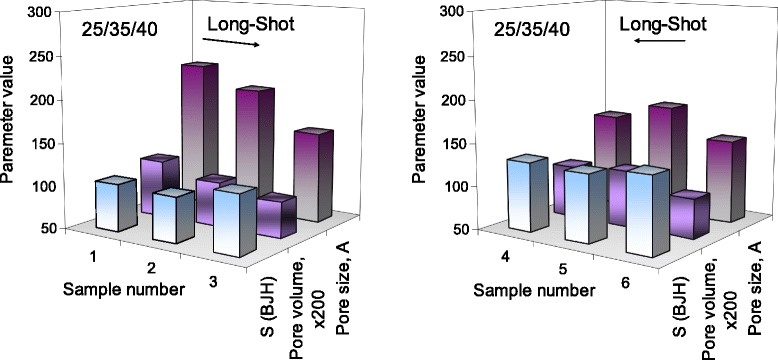
Diagram of a comparison of morphological parameters of the modified silica samples. The diagram shows the specific surface area (BJH and t-plot), pore size, and pore volume of samples synthesized by protocol 1 (samples 1–3, vol.% of the modifying mixture at the each stage 25/35/40) and protocol 2 (samples 4–6, vol.% of the modifying mixture at the each stage 40/35/25) that are located according to the scheme “from long to short” (L-S): the ratio of PMPS/DMC in the modifying mixture at the different stages of modification was 20/5.8/3.4, respectively (at the first modifying step the long-chain oligomers are introduced to the reactor)

According to the adsorption data for the samples obtained by protocol 1, the specific surface area is increased with a decrease in the length of oligomer in the modifying mixture used at the first modification step. Increase in specific surface area indicates increasing in a degree of dispersion of the material. Among samples 1–3 with specific surface area increase, the volume and pore size decrease (Fig. 4), indicating a convergence of the silica particles and densification of the agglomerates. The compaction of individual primary particles in the powder results in enhancement of particle–particle interactions. .

**Fig. 5:**
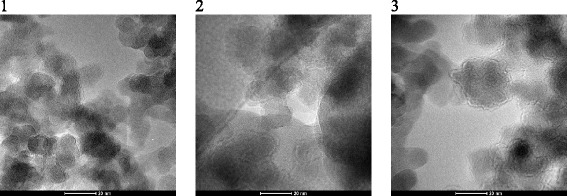
TEM images (*1*–*3*, the *scale bar* is 20 nm) of composites based on silica with immobilized PMPS using protocol 1. The photos illustrate the morphology of silica powders modified with PMPS by applying the protocol 1 (samples 1–3, the modifying mixture distribution at each stage was 25/35/40 vol.% of the total amount) that are synthesized according to the schemes: “from long to short” (L-S) *1*, “middle”(M) *2*, and “from short to long” (S-L) *3* (the ratio of PMPS/DMC in the modifying mixture at different stages of modification was 20/5.8/3.4, 5.5/5.5/5.5, and 3.4/5.8/20, respectively) (Table 1)

On the TEM image of samples 1–3 (Fig. 5) together with the native particles in large agglomerates, the individual particles with size of 20–30 nm are observed. Increasing the degree of dispersion of the material is confirmed by AFM data. For the samples obtained by protocol 1 (samples 1–3, the modifying mixture distribution on each stages was 25/35/45 vol% of the total amount) with increasing concentration of the depolymerization agent (DMC) in the first stage of the process a size of globules is decreased..

Consequently, the first stage of modification with oligomers that have less long chains promotes a destruction of primary aggregates of silica particles. Perhaps during modification, the shorter oligomers (sample 3) penetrate the interparticle space of primary agglomerates and part them, thereby increasing the availability of the active surface sites for contact with the polymer. In the next step, the longer oligomers complete the modifying layer by forming a shell around small secondary structures.

Upon the modification at the first stage with a small (relative to other stages) amount of long-chain oligomers, an interaction with particles is realized on the external agglomerates surface with formation at the next stages secondary polymeric layer rendered over the surface (Figs. 1 and 5).

According to the results of adsorption among nanopowders obtained by protocol 2, i.e., increasing the proportion of modified mixture in the first stage (40, 35, 25 % of total mixture volume on each stage), a significant effect of oligomers length immobilized at the beginning of the process was not observed (samples 4–6). At the same time, the specific surface area was higher than that for samples obtained by protocol 1. Changes in the length of introduced oligomers from short to long result in the nonlinear change in specific surface area. Increase in the specific surface area (SSA) does not lead to a decrease in pore volume. While maintaining the specific surface area, a large pore volume is coursed by an increase in a fraction of smaller pores. Among samples 4–6, the composite obtained using the scheme S-L (the silica modified in the first step by short-length oligomers) (Fig. 4) has the largest surface area. The smallest SSA was detected for the material modified with oligomers with equal length at the each stage (sample 5).

**Fig. 6:**
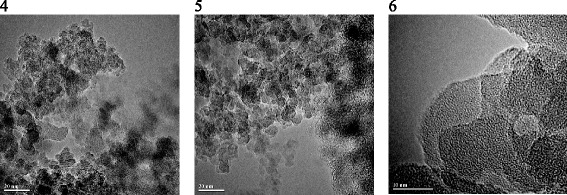
TEM images (*4* and *5*, the *scale bar* is 20 nm; *6*, the *scale bar* is 10 nm) of composites based on silica with immobilized PMPS using protocol 2. The photos illustrate the morphology of silica powders modified with PMPS by applying protocol 2 (samples 4–6, the modifying mixture distribution at each stage was 40/35/25 vol.% of the total amount) that are synthesized according to the schemes: “from short to long ” (S-L) *4*, “middle”(M) *5*, and “from long to short ”(L-S) *6* (the ratio of PMPS/DMC in the modifying mixture at different stages of modification was 20/5.8/3.4, 5.5/5.5/5.5, and 3.4/5.8/20, respectively) (Table 1)

This suggests that there is an optimum length of the kinetic segment of chain, providing penetration of the polymer into the space between the particles in prime units and provides a more complete polymer/particle contact. Meanwhile, for sample 5, with the specific surface area comparable to that of sample *3*, a larger pore volume at the highest pore size in the series of samples *4*–*6* was marked, as in the case of sample *1*. Therefore, the polymeric layer formed on the silica surface prevents particles compacting. This may suggest differences in the architecture of the grafted layer. Nevertheless, an increase in the amount of the modifying mixture with relatively long oligomers at the first step of modification (sample *6*) leads to compressing powder structure (reducing the pores size with their volume reduction in comparison with sample *1*), while increasing the quantity of the modifying mixture with the same oligomers length (sample *5* compared to sample *2*) leads to a reduction in pore size with the increase of their volume. On the TEM image of the samples obtained by the *protocol* 2 (Fig. 6), regardless of length of the oligomer introduced at the first stage, the contours of the primary silica particles are indistinguishable..

This may indicate a strong polymer/particles interaction and formation of the secondary structures with a high degree of homogeneity during synthesis.

**Fig. 7:**
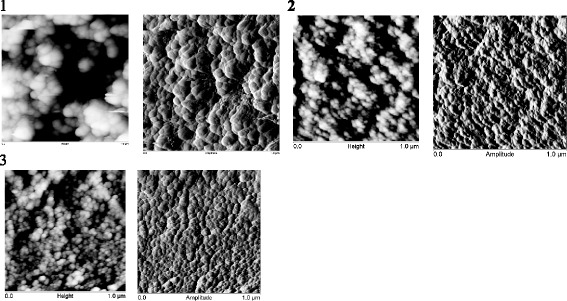
AFM scans of composites based on silica with immobilized PMPS using protocol 1. The photos illustrate the morphology of silica tablets composed from powders of SiO_2_ modified with PMPS by applying the protocol 1 (samples 1–3, the modifying mixture distribution at each stage was 25/35/40 vol.% of the total amount) that are synthesized according to the schemes: “from long to short” (L-S) *1*, “middle”(M) *2* and “from short to long” (S-L) *3* (the ratio of PMPS/DMC in the modifying mixture at different stages of modification was 20/5.8/3.4, 5.5/5.5/5.5 and 3.4/5.8/20, respectively) (Table 1)

**Fig. 8:**
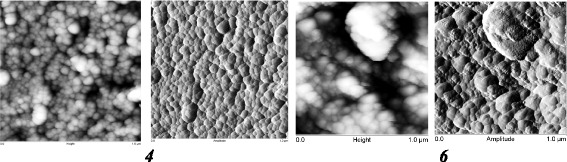
AFM scans (height and amplitude interval is 0.0–1.0 μm) of composites based on silica with immobilized PMPS using protocol 2. The photos illustrate the morphology of silica powders modified with PMPS by applying protocol 2 (samples 4 and 6, the modifying mixture distribution at each stage was 40/35/25 vol.% of the total amount) that are synthesized according to the schemes: “from short to long ” (S-L) *4* and “from long to short” (L-S) *6* (the ratio of PMPS/DMC in the modifying mixture at different stages of modification was 20/5.8/3.4 and 3.4/5.8/20, respectively) (Table 1)

Analysis of the XPS spectra showed that for samples *5* and *6*, the Si2p peak is described by one component related to the structural silicon. As for the samples obtained by protocol 1, for samples 4–6, the dispersity of material is decreased in series *6*-*5*-*4* with an increase in length of the oligomer introduced at the first stage of modification. However, the size of globules (AFM) is significantly higher (Figs. 7 and 8). Based on AFM images, a large pore volume can be caused by the presence of a hollow between large and small globules which provides access to inside globular pores..

On the basis of the textural characteristics (Tables 3, Figs. 3, 4, 5, 6, 7, and 8), one can assume that the structures of secondary particles of modified silicas are closely related to features of the structure of attached polymer on the nanoparticles surface that promote different *S* values. This is in agreement with TEM and AFM images of silicas obtained with applying different protocols of modification that demonstrate transformation morphology. The shape of nitrogen adsorption–desorption isotherms (Fig. 3a) corresponds to the same type II of the conventional classification of isotherms [32]. It suggests that there is similarity in the texture of obtained silicas with different specific surface area.

However, for composites 1–3, the N_2_ absorption ability *V*_p_ (pore volume) (Table 3) becomes higher with decreasing *S* value. According to [30], for the pristine silicas with different specific surface area (A-500, A-50), in a series of samples with a decrease in the specific surface area, narrowing of the hysteresis and increasing the diameter of the mesopores are noted that relate to a change in the size of the agglomerates, i.e., the size of aggregates becomes smaller, which results in an increase in the bulk density of agglomerates. Therefore, changing the length of the oligomer at the first stage provides control of the size of the secondary agglomerates (for individual particles it is observed at the less SSA and not visible in larger SSA), and modifying layer on the boundary of agglomerates prevents its agglutination, providing high values of *V*_p_.

For samples 4–5 at a high specific surface area, the individual particles are not distinguishable that corresponds to the data given in [30]. However, with the increase in specific surface area, the pore size and volume are decreased. Therefore, increasing the amount of modifying mixture at the first stage provides formation of a compact secondary structure, and the largest SSA (as opposed to samples 1–3) is achieved with the introduction of the first stage of long-chain oligomers.

It is known that the adsorbed linear PMPS (by analogy with PDMS) can form loops on the silica surface at the polymer content more than 8.8 % [33, 34]. However, polymer immobilization at the surface layer significantly reduces the mobility of the siloxane bridges and therefore prevents the formation of loops, as was shown in the study of degradation of PMPS adsorbed on the silica surface [26]. According to obtained results, even at 40 % polymer content, ring structures were detected in trace amounts (in contrast to similar studies of polydimethylsiloxane). Accordingly, the structure of the attached polymeric layer in the samples under study is preferably brush-like therefore and depends on the oligomers packing density adsorbed at the first step of modification.

Therefore, under applied synthesis conditions, the greater amount of modifying mixture introduced at the first stage provides a greater contact degree with surface (sealing layer), whereas the length of the oligomer introduced the distance from the surface on which the second layer is formed involving grafted oligomers. Under these conditions, the formation of relatively large aggregates with loosened structure, in which the particles probably contact through grafted long oligomeric chains, occurs.

**Fig 9:**
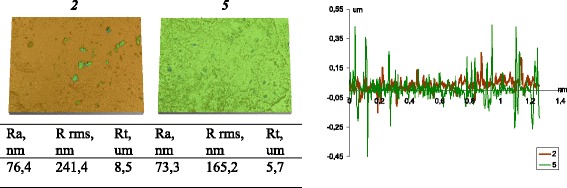
Roughness of the pellet of silica modified with PMPS using different protocol of synthesis. Figures show diagram and profile of pellet roughness for silicas obtained using protocol 1 (sample *2* obtained via scheme “M”; the PMPD/DMC ratio was equal to 5.5 at each stage, ACA of 121°), and protocol 2 (sample *5* obtained via scheme “M”; the PMPD/DMC ratio was equal to 5.5 at each stage, ACA of 131°). Studies were conducted on tablets compressed using the pressure 50 kgf/cm^2^ (Table 1)

Thus, the conditions of the grafted polymer layer formation are determined not only by the architecture of the surface layer but by a morphology of secondary structures. Figure 9 shows the results of measuring the surface roughness of the composite layer with light scattering spectroscopy. For samples with equal length of oligomers introduced at each stage of the modification (samples 2 and 5), depending on the amount of modifying mixture at the first step, a roughness was radically changed (Fig. 9).

**Fig. 10:**
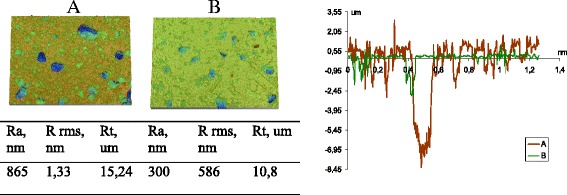
Roughness of the pellet of silica modified with PMPS by single-stage modification. Figures show a diagram and profile of the pellet roughness for modified silica obtained via single-stage process using mixture with a PMPS/DMC ratio 2/1 at 200 °C **a** (carbon content of 6.0 wt.%, contact angle −130° [39]) and 250 °C **b** (carbon content of 6.6 wt.%, contact angle −129° [39]). Studies were conducted on tablets compressed using the pressure 50 kgf/cm^2^

In both samples, the tablet surface is non-uniform (Fig. 9). However, the height profile is significantly different. If the lower quantities of the modifying mixture are introduced at the first step of modification, the surface of tablets obtained is smoother. At single-stage modification under the same conditions (temperature 250 °C, the degree of modification is 20 %, the ratio of polymer/DMC is 5.5), the sample loses uniformity. On the surface of the tabletcoverns are observed (Fig. 10b). The heterogeneity was decreased with temperature of the process increasing (Fig. 10a). However, the high degree of uniformity was achieved only at the multistep process.

### Hydrophilic–hydrophobic Properties of Composites

PMPS molecules can form a helix structure due to the corresponding rotations around the Si–O bonds [35, 36] that depends on the chain length and quantity of polymer at silica surface layer. In the immobilized state, the polymeric chains loose the mobility, and therefore, only the ends or/and portion of the segments of PMPS molecules can interact with the silica surface, e.g., by the formation of the hydrogen bonds ≡ SiO–H···O(Si(Ph)(CH_3_)–)_2_ or ≡ Si–O–Si(Ph)(CH_3_)–. As it was shown above, the structure of the adsorption/chemisorption complexes of the PDMS molecules depends on the conditions of the subsequent treatment. According to [37, 38], in concentrated solutions of siloxanes the fraction of unfolded molecules is increased that can result in an increase in the density of direct contacts between the PMPS molecules and the surface OH groups. As it was shown in [35], under these conditions, attachment is mainly realized by the coverage of whole aggregates and only little fraction of PDMS can penetrate into voids between primary particles in aggregates. The coating degree and distribution of molecules have an effect on hydrophilic/hydrophobic properties of particles.

The silica/PMPS composites were characterized by larger advancing (ACA) and receding (RCA) contact angles values (Table). The apparent surface free energy was calculated from the ACA and RCA values.

For samples under study, the ACA and RCA values is in the range of 117°–146°. At protocol 1, a high hydrophobicity was observed for sample modified at the first stage with long-chain oligomers (sample 1). Increase in the amount of modifying agent (protocol 2) at the same dosing regimes (scheme L-S) provides a significant increase in the surface hydrophobicity (sample 6). When added at the first stage short oligomers on both protocols (at considerable increase in the specific surface area), the composite hydrophobicity was reduced. For both series (samples 1–3 and sample 4–5), strong dependence of the hydrophobicity and the specific surface or carbon content (C%) is not observed (Table 4), indicating the determining role of the morphology of the resulting material.

**Table 4:** Advancing (ACA) and receding contact angles (RCA) of water, apparent surface free energy and the specific surface area (SSA)

No.	Scheme of modification	Advancing contact angle *θ*_a_ (deg.)	Receding contact angle *θ*_a_ (deg.)	Apparent surface free energy *γ*_S_ (mJ/m^2^)	SSA (BET)	^a^Carbon content (CNH). wt %	Carbon content (XPS). wt %
Protocol 1 (modifying mixture portion on tree stages—25/35/40 vol%)							
1	L-S	134	130	10	126	8.9	10.5
2	M	121	109	15	118	9.1	11.2
3	S-L	109	94	21	133	7.5	8.9
Protocol 2 1 (modifying mixture portion on tree stages—40/35/25 vol%)							
4	S-L	125	118	14	154	9.6	11.2
5	M	131	120	11	139	9.2	10.9
6	L-S	146	136	5	146	9.3	11.0

^a^Carbon content evaluated using CNH-analysis

According to the obtained results, a high hydrophobicity was determined for silica with relatively large agglomerates with high specific surface area (sample 6). Therefore, to achieve high hydrophobicity parameters, the architecture of grafted polymer layer is important, because it causes the morphology of secondary structures. The PMPS layer can be in contact not only with a surface of a particle (at the “open” surface of aggregates) but also with the surfaces of several particles that can change the aggregates size and morphology as well as strength of interaction and distance between adjacent oxide nanoparticles in aggregates. For samples under study, the powder texture of PMPS/silica composites remained mostly similar to that of the initial oxide suggesting well distribution of PMPS molecules at the surfaces of all nanoparticles. This is due to stronger interactions of PMPS with the oxide surface than in the case of PMPS–PMPS interactions. And, as it was shown above, the interaction of PDMS with the oxide surface can be stronger at smaller C(PMPS) values at the first modification stage with weaker lateral PMPS–PMPS interactions, that correlated with [35]. But with applying protocol 2, the PMPS–PMPS interaction becomes more intensive and promotes changes in the organization of aggregates and agglomerates. As reported in [35], the polymeric layer with lager molecular weight is more hydrophobic that suggests long polymeric chains to form at surface layer of composites, modified by protocol 2, from the other hand, high hydrophobicity is obtained for nanoparticles with monolayer covering. Literature data and obtained results suggest the formation in sample 6 hierarchic structure with unfolded spread from surface polymeric molecules that interact with modified layer of adjacent particles.

It was shown that uniform morphology with small agglomerates causes a smooth topography with remained hydrophilicity of composites despite the hydrophobic properties of PMPS. So, the hydrophobicity depends not only the surface chemistry but also on the surface topography (roughness). An increase in the surface roughness with an increase in the modifying agent concentration at the first stage of the process (sample *5* in comparison with sample *2*) causes an increase in the hydrophobicity and correlates with the literature data. However, for shorter oligomers introduced at the first modification stage, the morphology of surface layer is mostly linear and may be due to displacement of shorter oligomers with longer ones at the next modification stage, or due to covering of first immobilized layer by longer oligomers by following modification, the morphology features are remained similar and composites had uniform structure but low hydrophobicity.

## Conclusions

Changes in synthesis conditions allow one to vary the structure of contacts between adjacent primary particles in their aggregates, which affect the structural and adsorption properties of the powders. Different types of treatment of the silica allow one to change PMPS–particle interactions, which results in variation of the layer structure.

The results indicate a significant effect of the PMPS/DMC ratio at the each modification stage on hydrophobic properties of modified silicas. Modifying with a similar composition of the PMPS/DMC mixture, even with different polymer amount at the each stage, provides the worst hydrophobicity. Results suggest that the highest hydrophobicity is achieved in the case of silica modified with the mixture providing the formation of monomolecular layer of polysiloxane at the surface during the first step of the process and building of long-chain hierarchic structures at the next modification steps that promotes interaction between nanoparticles in aggregates at relatively large distance and formation of the agglomerates with high roughness. The obtained results will be particularly useful for forming surface layers and polysiloxane coating with super hydrophobic properties; such materials are of particular interest in the development of protective and self-cleaning coatings, water-repellent materials, and biological gels.

## Abbreviations

ACA, advancing contact angles; AFM, atomic force microscopy; CNH, carbon-oxygen-nitrogen content analysis; D, pore diameter; DMC, dimethyl carbonate; FTIR, fourier transform infrared spectroscopy; PMPS, polymethylphenysiloxane; RCA, receding contact angles; SSA, specific surface area; TEM, transmission electron microscopy; *V*_p_, pore volume; XPS, X-ray photoelectron spectroscopy
